# Regional Gray Matter Volume Is Associated with Empathizing and Systemizing in Young Adults

**DOI:** 10.1371/journal.pone.0084782

**Published:** 2014-01-07

**Authors:** Hikaru Takeuchi, Yasuyuki Taki, Yuko Sassa, Hiroshi Hashizume, Atsushi Sekiguchi, Ai Fukushima, Ryuta Kawashima

**Affiliations:** 1 Smart Ageing International Research Center, Institute of Development, Aging and Cancer, Tohoku University, Sendai, Japan; 2 Division of Developmental Cognitive Neuroscience, Institute of Development, Aging and Cancer, Tohoku University, Sendai, Japan; 3 Department of Functional Brain Imaging, Institute of Development, Aging and Cancer, Tohoku University, Sendai, Japan; Centre Hospitalier Universitaire Vaudois Lausanne - CHUV, UNIL, Switzerland

## Abstract

Empathizing is defined as the drive to identify the mental states of others for predicting their behavior and responding with an appropriate emotion. Systemizing is defined as the drive to analyze a system in terms of the rules that govern the system in order to predict its behavior. Using voxel-based morphometry and questionnaires in a large sample of normal, right-handed young adults, we investigated the regional gray matter volume (rGMV) correlates of empathizing and systemizing and additionally those of the D score, which is the difference between systemizing and empathizing, to reveal the comprehensive picture of those correlates. Negative rGMV correlates of empathizing and positive rGMV correlates of the D score (formed by the negative correlation between rGMV and empathizing), were found primarily in nodes in the default mode network, mirror neuron system, dorsal anterior cingulate cortex, and the lateral part of the prefrontal cortex together with other areas. Positive rGMV correlates of systemizing and of the D score (formed by the positive correlation between rGMV and systemizing) were found primarily in nodes in the external attention system, middle cingulate cortex, and other regions. Negative rGMV correlates of systemizing were found in an area close to the left posterior insula and putamen. These findings reconcile some previously inconsistent findings, provide other new findings and suggest that these areas contribute to empathizing–systemizing. Furthermore, the negative/positive rGMV correlates of empathizing and positive/negative rGMV correlates of systemizing overlapped substantially. This may be in line with the notion that empathizing and systemizing compete neurally in the brain.

## Introduction

Empathizing is defined as the drive to identify the mental states of others for predicting their behavior and responding with an appropriate emotion [1]. Systemizing is defined as the drive to analyze a system in terms of the rules that govern the system in order to predict its behavior [1]. Growing evidence shows that males exhibit greater systemizing than females, whereas females exhibit greater empathizing than males [2]–[4]. Moreover, the empathizing–systemizing theory posits that autism spectrum conditions (ASCs), such as Asperger’s syndrome, represent an extreme disposition toward reduced empathizing and enhanced systemizing [1].

The systemizing quotient (SQ) and empathizing quotient (EQ) questionnaires [3], [5], developed to evaluate individual differences of empathizing and systemizing, are self-report questionnaires. However, their validity in quantifying empathizing–systemizing has been established. Individuals with ASCs, males, and science students have higher SQ scores and lower EQ scores [2], [4]. A higher EQ score is also associated with actors [6], larger individual social networks [7], and performance on a face perception task [8]. The Autism Spectrum Quotient (AQ), a measure of autistic traits, is well explained by a model including both EQ and SQ [9]. Furthermore, the D score, the difference between systemizing and empathizing, better describes ASCs and differentiates typical males from typical females than EQ and SQ alone [10]. However, the sum of the empathizing and systemizing scores does not differ between males and females [11]. On the basis of this evidence, it has been suggested that empathizing and systemizing compete neurally in the brain and that the D score provides information on the trade-off between the two [11]. However, other mechanisms may lead to the same sum of empathizing and systemizing between males and females. Moreover, little correlation exists between empathizing and systemizing [4], [9]; therefore, this idea has been criticized and remains controversial [12].

The empathizing–systemizing theory posits that empathizing–systemizing can explain many behavioral and cognitive features of individuals with ASCs, such as inferior social, emotional, and empathetic skills [13], [14]. These individuals have a reduced theory of mind (TOM) compared with those without [15]. TOM has been suggested as a key component of empathizing [16], and it is critical for detecting information related to the mental states, emotions, and intentions of others and for recognizing how the feelings of others may impact an individual [17]. Studies have shown that individuals with ASCs have enhanced abilities in math, physics, and engineering, all of which require a high level of systemizing [1], and enhanced spatial abilities that require systemizing in various ways, such as focusing on the details of an object and predicting how it can be transformed [1].

Two recently identified intrinsic brain networks, the default mode network (DMN) and the external attention system (EAS) [18], [19], may be associated with empathizing–systemizing. DMN regions are active at rest, while they are usually suspended during externally directed, attention-demanding tasks. Regions of EAS, which is dedicated to external attention, respond to these tasks in the opposite manner [18]–[20]. Regions such as the medial prefrontal cortices (mPFCs), precuneus, and superior temporal sulcus (STS) belong to DMN, while regions such as the lateral PFC, inferior parietal lobe, dorsal anterior cingulate cortex (dACC), supplementary motor area, and temporoparietal cortex belong to EAS [18]–[21]. Furthermore, DMN regions are activated by various cognitions for which individuals with ASCs show inferiorities, including TOM, and the recognition of another’s perspective [18], [22]. However, empathy is a complex phenomenon and recruits a wide range of regions. Lesion and functional activation studies related to individual differences in empathy have consistently shown that mPFC, which is the key DMN node, plays a key role in individual differences in empathy [23]–[29]. On the other hand, EAS is divided into the dorsal attention network and the ventral attention network [30]. EAS works in opposition to DMN [21] but in association with other networks, such as the salience network [31]. Furthermore, EAS regions are activated by various cognitions, in which ASCs show superiority, or which are related to systemizing. These include spatial cognition [32], attention to detail [33], [34], and modus tollens reasoning (“if p, then q”) [33], [34]. Furthermore, mathematicians have higher regional gray matter (rGM) density in EAS regions [35], and a functional imaging study has shown that individual systemizing is associated with functional activation of EAS regions [36]. However, the functions of the intrinsic network and of regions belonging to this network may differ; therefore, to the best of our knowledge, the idea that these functions are identical remains an assumption.

Much attention has been paid to empathizing–systemizing, and some studies have investigated the association between functional activity and empathizing–systemizing [36], [37]. Lesion and GM structural studies of empathy have been conducted in patients with neurodegenerative diseases [23]–[25]. However, despite being strong, findings from the lesion studies may suffer from poor spatial resolution and an inability to investigate whole brain structures. Furthermore, structural correlates of certain cognitive functions may differ among patients with certain diseases and normal individuals [38] and among patients with different diseases [25]. The strength of structural imaging is that the results are not constrained by a specific functional task performed in a scanner. Thus, observing structural correlates of empathizing–systemizing in normal individuals can provide useful and distinctive information.

Anatomical correlates of empathy-related measures, systemizing, and the D score have previously been studied in normal populations [39]–[44]. **Table 1** summarizes the characteristics and results of these studies. However, several issues remain to be resolved. First, anatomical correlates of empathy reported in these studies have been inconsistent, which may be due to several factors including differences in methodology {subject characteristics, preprocessing, scale, and regions of interest (ROIs)} and relatively small sample sizes that result in insufficient statistical power. As noted earlier, empathizing is supposed to underlie a wide range of cognitions and many regions can contribute to empathizing, thus revealing all of these may require great statistical power. Second, in adults, the association between rGMV and systemizing (and also the D score) has only been investigated in males [40]. Thus, given that the empathizing–systemizing theory has tended to focus on sex-related differences, whether there are sex-related differences in rGMV correlates of systemizing remains to be determined. Furthermore, a very lenient cluster-determining threshold was used in the study by Lai et al. [40], and regardless of the cause, they identified rather large clusters. Because the cluster test cannot identify where exactly the significant effects of interest are in such large clusters [45], when the identified clusters are too large, it cannot localize the findings very well. Thus, more localized anatomical correlates of systemizing in normal adults remain undetermined.

**Table 1:** Summary of previous gray matter structural studies of empathetic scales and systemizing and relevant measures in normal samples.

	Psychologicalmeasure	Subjects	GMmeasure	Areas
				Previous results related to empathy or socialityrelated to autistic traits
Banissy et al. [42]	EC (IRI)[127] *	118 healthy adults (66 females, mean age: 22.9 years, SD: 4.2 years)	rGMV	Negative correlation in the dACC, left insula, leftIFG, and precuneus
Banissy et al. [42]	PT (IRI)[127] *	Same as the above	rGMV	Positive correlation in dACC
Cheng et al. [39]	ECS[128]	50 healthy adults (25 females, mean age: 27.1 years, range: 19–49 years)	rGMV	Positive correlation in the right IFG
Cheng et al. [39]	EETS[129]	Same at the above	rGMV	Positive correlation in the right IFG and rightinferior parietal lobule
Cheng et al. [39]	EC(IRI)*	Same as the above	rGMV	Positive correlation in the right IFG, right inferiorparietal lobule, and mPFC
Cheng et al. [39]	EQ	25 healthy males (mean age: 26.6 years, range: 19–49 years)	rGMV	Positive correlation in the right IFG and mPFC
Mutschler et al. [43]	E–Sscale [130]	101 healthy females (mean age: 23.6 years, range: 13–35 years)	rGMD	Positive correlation in the left anterior insula
Sassa et al. [41]	EQ	290 healthy children (145 females, mean age: 10.6 years, range: 5.6–18.4 years)	rGMV	Positive correlation in the cluster that includesthe left IFG, superior temporal gyrus, precentralgyrus, and middle to posterior insula.
Wallace et al. [44]	SRS[131]	323 typical (malinly) children (147 females, mean age: 10.6 years, SD: 3.7 years)	Cortical thickness	Negative correlation in the bilateral superiortemporal gyrus, bilateral middle temporal gyrus,right angular gyrus, left superior parietal lobule,and precuneus
				Previous results related to systemizing (and Dscore)
Sassa et al. [41]	SQ	290 healthy children (145 females, mean age: 10.6 years, range: 5.6–18.4 years)	rGMV	Negative correlation in the cluster that includesthe left posterior parietal cortex and precuneus.
Lai et al. [40]	SQ	88 normal males (mean age: 29 years, range: 18–45 years)	rGMV	Positive correlation in the cluster of the median GM structures (dorsal mPFC, ACC, middle cingulate cortices, and SMA)Negative correlation in the bilateral clusters thatincluded the left hypothalamus, posterior insula,nucleus accumbens, caudate, putamen, andpallidum (only the left one included the insula**)
Lai et al. [40]	D score	Same as the above	rGMV	Positive correlation in the cluster of the median GM structures (dorsal medial prefrontal areas, anterior and middle cingulate cortices, and SMA)Negative correlation in the bilateral clusters that included the left hypothalamus, posterior insula, nucleus accumbens, caudate, putamen, and pallidum (only the left one included the insula**)

Previously reported rGM structural correlates of empathetic competence or relevant measures include the mPFC, dACC, IFG, insula, precuneus, inferior and superior parietal lobules, superior and middle temporal gyri, precentral gyrus, and angular gyrus. Those for systemizing include the median GM areas, subcortical areas, precuneus, and posterior parietal cortex.

IRI has two other subscales, distress and fantasy. In accordance with Rankin et al.’s [25] contention that these two subscales do not measure empathetic competence well, they were not included in this table.

The inclusion of the insula in the cluster was confirmed by the cordial offer of the mask for the clusters by the authors.

Abbreviations: dACC, dorsal anterior cingulate cortex; EC, empathetic concern from the Internal Reactivity Index; ECS, emotional contagion scale; EETS, emotional empathic tendency scale; EQ, empathizing quotient; GM, gray matter; IFG, inferior frontal gyrus; IRI, Internal Reactivity Index; mPFC, medial prefrontal cortex; PT, perspective taking from the Internal Reactivity Index; rGMD, regional gray matter density; rGMV, regional gray matter volume; SD, standard deviation; SMA, supplementary motor area; SQ, systemizing quotient; SRS, social responsiveness scale.

Growing evidence suggests that ASCs exist on a continuum with normality [5] and that empathizing and systemizing are normally distributed in normal populations [1]. This suggests that studying correlates of empathizing and systemizing in normal populations is useful and has indeed been the case in psychological and neuroimaging studies. Several correlates of empathizing–systemizing have been investigated in typically developing young adults through these studies. Thus, investigating these correlates in typically developing young adults is important. The study aimed (a) to integrate previously reported inconsistent findings relating to rGMV correlates of empathy/empathizing using a large sample; (b) to identify localized anatomical correlates of systemizing; and (c) to identify sex-related differences in the rGMV correlates of empathizing and systemizing by investigating the correlates in a large sample of typically developing young adults. We additionally investigated those issues for the D score. We hypothesized that rGMV in regions associated with DMN and EAS would be associated with empathizing and systemizing, respectively. Specifically, we hypothesized that the key nodes of DMN and EAS, i.e., mPFC/precuneus/STS and the lateral PFC, would be associated with empathizing and systemizing, respectively. To address inconsistencies in the previous study findings, we recruited a large sample (567 subjects; 329 men and 238 women) of individuals of a specified age (20.8±1.9 years).

## Materials and Methods

### Ethics Statement

In accordance with the Declaration of Helsinki (1991), written informed consent was obtained from each subject. This study was approved by the Ethics Committee of Tohoku University.

### Subjects

Five hundred and sixty-seven healthy, right-handed individuals {329 men and 238 women; mean age, 20.8 years (standard deviation, 1.9)} participated. All subjects were college, university, or postgraduate students, or had graduated within the previous year. For more details and related discussions of limitations regarding the study participants, see **Methods S1** and **Discussion S1**.

### SQ–EQ Questionnaires

Japanese versions [4] of the SQ and EQ questionnaires [3], [5] were administered. EQ and SQ scores were used as indices of empathizing and systemizing, respectively. These tests consist of 40 items for each quotient and 20 filler items that are not scored. The following are examples of items found on the SQ–EQ questionnaires:

“I can tune into how someone else feels rapidly and intuitively” (EQ)

“I am good at predicting how someone will feel” (EQ)

“I am fascinated by how machines work” (SQ)

“If I were buying a stereo, I would want to know about its precise technical features” (SQ)

The questionnaires comprise self-descriptive statements scored on a four-point scale ranging from Strongly Disagree to Strongly Agree. Half the items are worded to produce an “agree” response and the remaining to produce a “disagree” response. The items are randomized to avoid a response bias. Each strong systemizing/empathizing response is awarded 2 points, and each slight systemizing/empathizing response is awarded 1 point (i.e., each item is scored as 2, 1, or 0), resulting in a total score of 0–80 for each quotient.

These scales are known to be reliable. The internal consistency of EQ and SQ among normal subjects has a Cronbach’s α coefficient of 0.86 and 0.88, respectively [4]. As noted earlier, the criterion-related validity of this questionnaire has been demonstrated, and individuals with ASCs have been shown to have higher SQ and lower EQ scores than those without [4]. Furthermore, males have higher SQ scores than females, while females have higher EQ scores than males [2]. For theoretical and practical issues relating to EQ and SQ, see **Discussion S1**.

The D score was calculated as described previously [11]. Raw SQ and EQ scores were standardized by subtracting the population mean from the score and then dividing the result by the maximum possible score: S = (raw SQ score − population mean of the raw SQ score)/80 and E = (raw EQ score − population mean of the raw EQ score)/80. For this computation, we used estimated population means (EQ: mean, 33.4; SQ: mean, 22.7) derived from a large sample (N = 1250) of Japanese university students in a previous study (which included an almost equal number of males and females) [4]. The discrepancy between systemizing and empathizing was then quantified as D = (S − E)/2. The greater the D score in a positive direction, the stronger one’s systemizing relative to one’s empathizing. D scores close to zero represent an equal drive to systemize and empathize. The D score is a measure widely used in research by leading experts in relevant areas [2], [4], [9], [11], [40], [46]. The score is better at distinguishing ASCs from controls and differentiating typical males and females [2], [4], [9], [11], predicting entry into physical sciences and humanities [2], [46], [47] and predicting programming aptitude [48] than EQ or SQ. However, because the D score has components of both S and E, examining correlates of the D score alone cannot reveal the whole picture. Thus, we also investigated the correlates of E and S scores. One of the problems with using the difference between two values is that when the difference is calculated, determining the source of variations of the value is not possible [49]. However, in the present study, the difference in SD of EQ and SQ scores was not substantial (EQ: SD, 9.63; SQ: SD, 8.44). Furthermore, z scores of the EQ and SQ scores can be used to calculate the D score [4], and this can control for differences in SD of the EQ and SQ scores. However, we used the present method to calculate the D score partly because it is more widely used [1], [11], [14], [50], [51] and partly because the distribution of the D score calculated using the z scores of EQ and SQ is very similar to that calculated using the present method and produced similar imaging findings [40].

### Assessment of General Intelligence

General intelligence refers to the *g* factor [52], which contributes to success on diverse forms of cognitive tests, regardless of whether these are verbal or non-verbal. Raven’s Advanced Progressive Matrix (RAPM) [53], which is the measure that is most correlated with *g* and is thus the best measure of general intelligence [53], was used to assess general intelligence. The test was used in the present study to adjust for the effect of individual psychometric measures of intelligence on brain structure. For more details on RAPM, see our previous studies [54], [55].

### Image Acquisition

MRI data acquisition was performed using a 3-T Philips Achieva scanner. High-resolution T1-weighted structural images (T1WIs: 240×240 matrix; TR, 6.5 ms; TE, 3 ms; FOV, 24 cm; slices, 162; slice thickness, 1.0 mm) were collected using a MPRAGE sequence.

### Preprocessing of the Morphological Data

Preprocessing of the data from T1WIs was performed using VBM2 software [56], an extension of SPM2. Default parameter settings were used [56].

We used a scanner-specific customized GM anatomical template and prior probability maps from GM and white matter (WM) images constructed from T1WI taken using the same scanner as that used in our previous study [55], because T1WI obtained in the present study may have differed from the existing template and because each scanner introduces specific non-uniformities in image intensity and inhomogeneities in the B0 field. T1WIs of each subject were segmented into GM and WM partitions using the abovementioned customized GM and WM prior probability maps [55]. The resulting images included extracted GM and WM partitions in the native space. The GM partition was then normalized to the abovementioned customized GM probability map from the previous study [55]. The normalization parameters determined from this initial step were then applied to the native T1WI. These normalized T1-weighted structural images were then segmented into GM and WM partitions. Moreover, we performed a volume change correction (modulation) by modulating each voxel using the Jacobian determinants derived from spatial normalization, allowing for the determination of regional differences in the absolute amount of GM [57]. This resulted in rGMV-representing maps. Subsequently, all images were smoothed by convolving them with an isotropic Gaussian Kernel of 12-mm full width at half maximum. For the reasons for using VBM2, see our previous studies [58]–[61]; basically, VBM5/VBM8’s preprocessing is not compatible with our T1-weighted structural images, and we cannot alter this.

### Statistical Design

The morphological data were statistically analyzed using VBM5 software [56], an extension of SPM5. These analyses only included voxels with GM of >0.05 to avoid possible partial volume effects around the borders between GM and WM and between GM and cerebral spinal fluid. We tested for relationships between rGMV and the EQ/SQ scores and attempted to determine whether any such relationships differed between the sexes. We also investigated these relationships for D score.

Whole-brain multiple regression analysis was used to test for relationships between EQ/SQ scores and rGMV. This analysis included six covariates: sex, age, RAPM score, total brain volume, EQ score, and SQ score. For the reasons for choosing these models, see **Discussion S1**. Next, we tested whether the relationships between rGMV and the EQ/SQ score differed between the sexes (i.e., whether any interaction between sex and the EQ/SQ score affected rGMV). In the whole-brain analysis, we used a voxel-wise analysis of covariance (ANCOVA) with the sex-related difference as a grouping factor (using the full factorial option in SPM5). Age, RAPM score, total brain volume, EQ score, and SQ score were used as covariates. All of these covariates, except the total brain volume, were modeled in ANCOVAs so that the unique relationship between each covariate and rGMV could be detected for each sex (using the interactions option in SPM5). This allowed us to investigate the interaction effects of sex and each covariate. We used t-contrasts to assess the interaction effects between (a) sex and the EQ score (in the second ANCOVA) and (b) sex and the SQ score (in the second ANCOVA) on the basis of rGMV values.

We also performed another whole-brain multiple regression analysis and another whole-brain ANCOVA in which EQ and SQ scores were replaced with the D score.

### Statistical Threshold

The significance level was generally set at *P<*0.05, corrected at the non-isotropic adjusted cluster level [62] with an underlying voxel level of *P<*0.0025. However, in the contrasts, where the cluster size test became theoretically inappropriate since the clusters formed were too big, a multiple comparison correction was performed using the false discovery rate (FDR) approach [63] as the second-best option. For details on the cluster size tests used and the selection of the present thresholding methods, see **Methods S1**. In these contrasts, we reported clusters that contained more than five voxels below the threshold of *P*<0.05, corrected for FDR.

Furthermore, the significance level was set at *P<*0.05 with the use of a small volume correction (SVC) for multiple comparisons (FDR) in ROIs for areas with a strong *a priori* hypothesis but without significant results in the whole-brain analyses. These areas included mPFC/precuneus/STS for the analyses of empathizing and the D score and the right lateral PFC for the analysis of systemizing and the D score. As described earlier, these regions are key nodes of DMN and EAS and the core of our hypotheses. Furthermore, the right dorsolateral PFC (DLPFC) and right ventrolateral PFC are consistently correlated with systemizing in different types of brain activities of interest [36]. The mask image comprised a sphere (12 mm radius; smoothing size) around peak voxels in mPFC.

The peak voxels in mPFC (*x, y, z* = −1, 47, −4) and precuneus (*x, y, z* = −5, −49, 40) were selected from a previous representative study of DMN [21]. The peak voxels in the bilateral STS (*x, y, z* = ±55, −55, 11) were selected from a previous study of autism spectrum traits (ASQ) in the normal population that focused on this region [64]. The peak voxels in the right DLPFC (*x, y, z* = 38, 39, 33) and the ventrolateral PFC (*x, y, z* = 50, 23, −1) were selected from the results of a previous functional imaging study on systemizing [36]. The peak voxels of these regions were selected on the basis of the representativeness of the previous study [21] or because of similar themes.

## Results

### Behavioral Data

**Table 2** shows the average score, standard deviation, age range, EQ/SQ/D score, RAPM score for each sex, and the statistical values for the comparison between males and females (two-tailed *t*-tests). **Table 3** presents simple correlation coefficients among the EQ/SQ/D and RAPM scores in all subjects, male subjects, and female subjects.

**Table 2:** Demographic variables for males and females and statistical results of the comparisons between males and females (two-tailed *t*-tests).

		Males			Females		*P* value	*t* value
Measure	Mean	Standard deviation	Range	Mean	Standard deviation	Range		
Age	20.89	1.94	18–27	20.78	1.82	18–27	0.544	0.61
RAPM	28.72	3.72	15–36	28.08	3.71	18–36	0.045	2.01
EQ score	29.69	9.63	9–66	35.11	9.40	14–63	5.36*10^−11^	−6.70
SQ score	27.76	8.44	9–56	21.62	7.11	8–45	1.82*10^−19^	9.37
D score	0.0548	0.0736	−0.152 to 0.298	−0.0174	0.0715	−0.221–0.192	2.41*10^−28^	11.73

RAPM, EQ, SQ, and D scores showed sex-related differences.

Abbreviations: RAPM, Raven’s Advanced Progressive Matrix; EQ, empathizing quotient; SQ, systemizing quotient.

**Table 3:** Simple correlation coefficients between psychological variables (and *P* values) in all subjects (MF), male subjects (M), and female subjects (F).

	EQ score	SQ score	D score	RAPM score
EQ score	–	–		–
SQ score	MF: 0.012 (0.780)	–	–	–
	M: 0.157 (0.004**)			
	F: 0.060 (0.353)			
D score	MF: 0.757 (2.36*10^−106^ ***)	MF: 0.645 (5.71*10^−68^ ***)	–	–
	M: 0.707 (6.95*10^−51^ ***)	M: 0.589 (4.52*10^−32^ ***)		
	F: 0.784 (7.97*10^−51^ ***)	F: 0.571 (4.48*10^−22^ ***)		
RAPM score	MF: −0.095(0.023*)	MF: 0.128 (0.002**)	MF: 0.156 (1.90*10^−4^ ***)	–
	M: −0.010(0.854)	M: 0.118 (0.032*)	M: 0.093 (0.092)	
	F: −0.168(0.009**)	F: 0.083 (0.200)	F: 0.190 (0.003**)	

*P*<0.05,

*P*<0.01,

*P*<0.001.

Abbreviations: RAPM, Raven’s Advanced Progressive Matrix; EQ, empathizing quotient; SQ, systemizing quotient.

### Correlation between rGMV and EQ/SQ Scores Across Sexes

**Figure 1:**
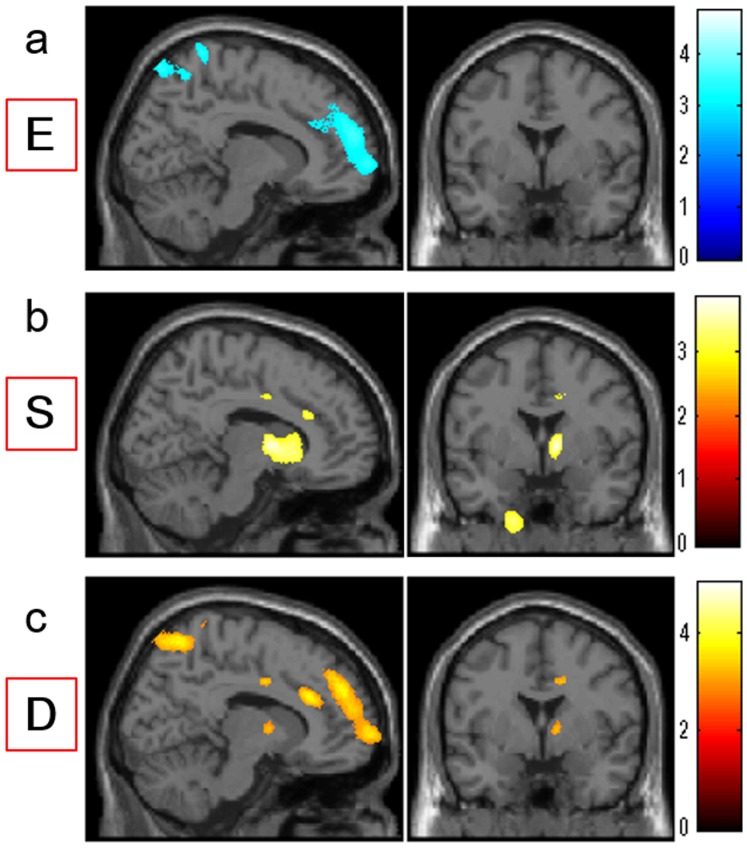
Regions with correlations between empathizing, systemizing, D score, and rGMV in or close to the lateral parts of the brain. (a) Regions with negative correlations between rGMV and empathizing. Results are shown with *P*<0.0025, uncorrected. Regions with correlations are shown in clusters, which were mainly located in a wide range of frontal and parietal areas. (b) Regions with positive correlations between rGMV and systemizing. Results are shown with *P*<0.0025, uncorrected. Regions with correlations are shown in the right middle frontal gyrus and other regions. (c) Regions with positive correlations between rGMV and the D score. Results are shown with *P*<0.0025, uncorrected. Regions with correlations are shown in clusters, which were mainly located in a wide range of frontal and parietal areas and in other regions.

**Figure 2:**
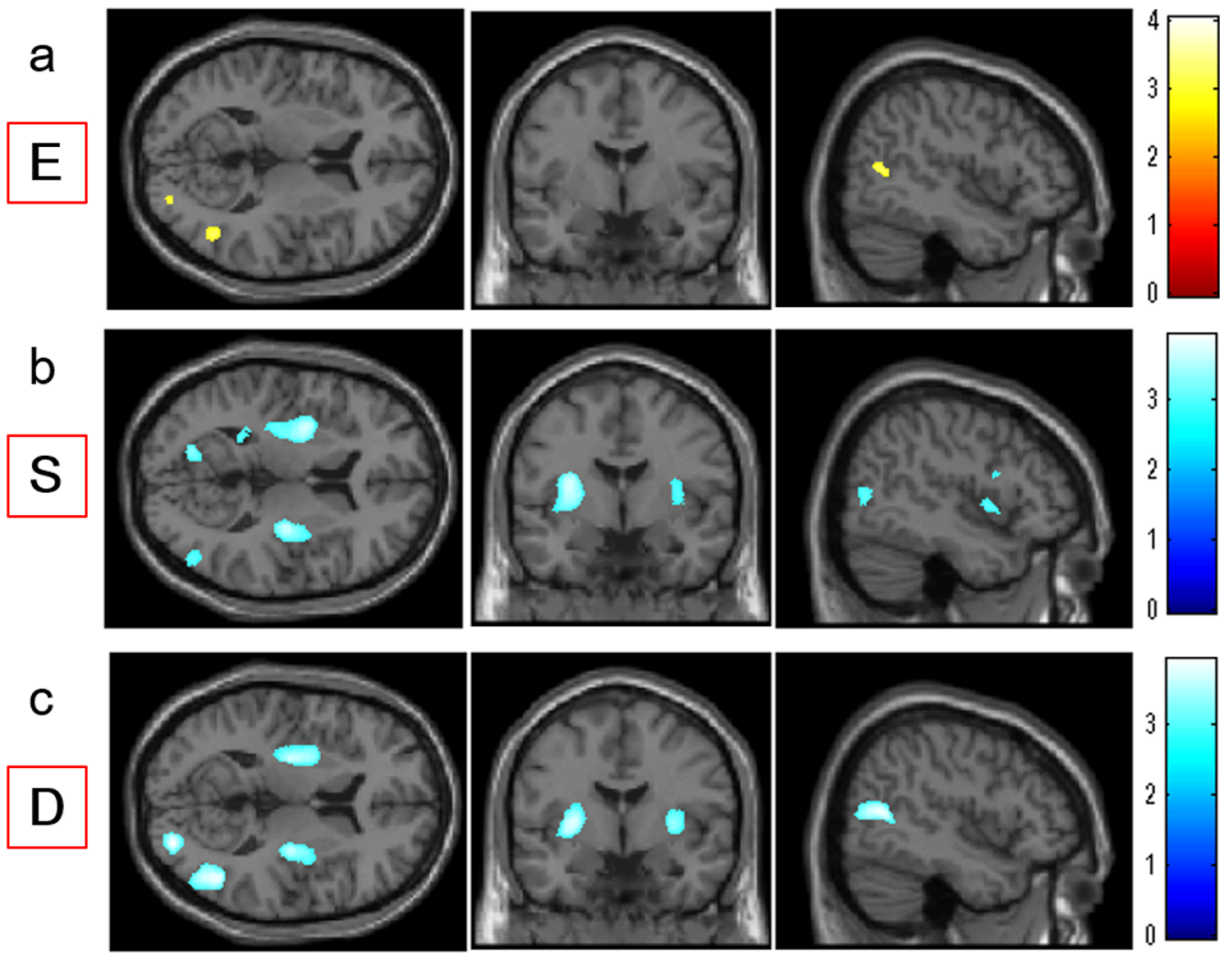
Regions with correlations between empathizing, systemizing, D score, and rGMV. (a), (b), (c) Regions with correlations are overlaid on a single subject T1 image of SPM5 in sagittal (left panels; x = 12) and coronal (right panels; y = 0) views. Results are shown with *P*<0.0025, uncorrected. (a) Regions with negative correlations between rGMV and empathizing. Regions with correlations are shown in clusters, which were mainly located in the medial prefrontal, anterior cingulate, and medial parietal areas. (b) Regions with positive correlations between rGMV and systemizing. Regions with correlations are shown in areas of dACC, middle cingulate cortex, and areas around the caudate and extranuclear regions. (c) Regions with positive correlations between rGMV and the D score. Regions with correlations are shown in the medial prefrontal areas, anterior cingulate cortex, middle cingulate cortex, medial parietal regions, and the extranuclear area close to the caudate.

**Figure 3:**
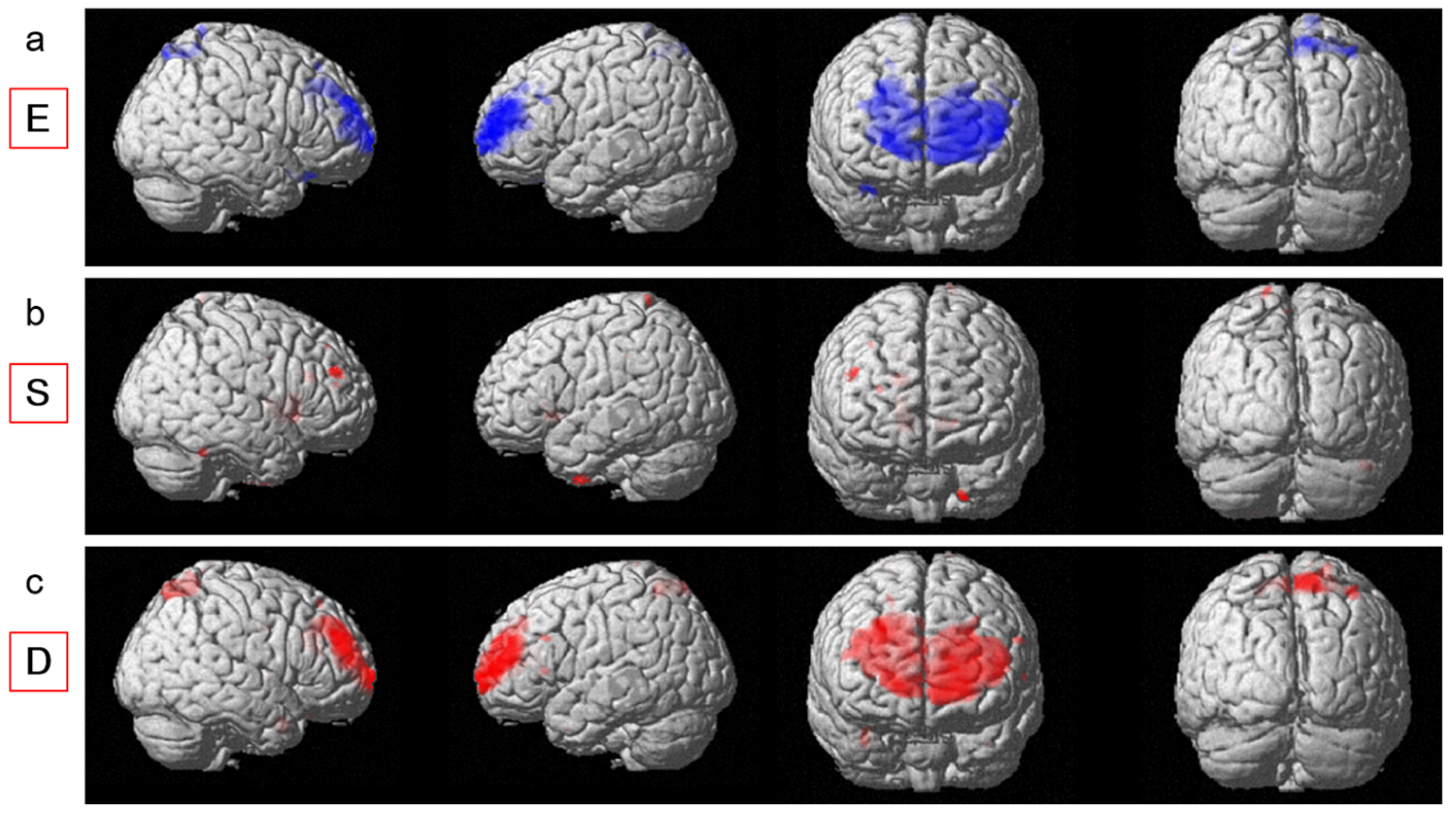
Regions with correlations between empathizing, systemizing, D score, and rGMV. (a), (b), (c) Regions with correlations are overlaid on a single subject T1 image of SPM5 in the axial (left panels; z = 47), coronal (middle panels; y = −63), and sagittal (right panels; x = 11) views. Results are shown with *P*<0.0025, uncorrected. (a) Regions with positive correlations between rGMV and empathizing. Regions with correlations are shown in the right STS. (b) Regions with negative correlations between rGMV and systemizing. Regions with correlations are shown in the right STS, a cluster that included the left posterior insula and left putamen, and other regions. (c) Regions with negative correlations between rGMV and the D score. Regions with correlations are shown in the right STS, a cluster that included the left posterior insula and left putamen, and other regions.

Multiple regression analysis, including age, sex, general intelligence, total brain volume, and SQ score, showed that the EQ score was significantly and negatively correlated with GMV in a large anatomical cluster that included regions of the bilateral mPFCs, bilateral superior frontal gyrus, middle frontal gyrus, left inferior frontal gyrus (IFG) and regions of the precuneus, right orbital frontal gyrus, right superior parietal lobule, medial frontal gyrus, and anterior cingulate gyrus (Figs. 1b, 2b, 3b). SVC using FDR were applied in ROIs for areas with a strong *a priori* hypothesis but without significant results in the whole-brain analyses. These areas included mPFC/precuneus/STS for the analyses of empathizing and the D score and the right lateral PFC for the analysis of systemizing and the D score. As described in Introduction, these regions are key nodes of DMN and EAS and the core of our hypotheses. For more details of the procedures and reasons, please refer to the subsection 2.4 of the Methods. But there were no other significant results in this contrast. Among these areas, as seen the subsection below, the rGMV in the right orbital frontal gyrus was not significantly positively correlated with the D score. As described below, rGMV in a region in the left IFG showed a significant negative correlation with the D score probably mainly as a result of a trend-level negative correlation with the EQ score (*x, y, z* = −52, 22, 28, *t* = 3.23, *P* = 0.053, corrected for FDR).

Multiple regression analysis, including age, sex, general intelligence, total brain volume, and EQ score, showed that the SQ score was significantly negatively correlated with rGMV in an anatomical cluster that included the left posterior insula and the left putamen at the whole-brain level. No other significant results were observed at the whole brain level, although a trend-level negative correlation was observed between rGMV and the SQ score in an anatomical cluster that included areas in the right posterior insula and right putamen (*x, y, z* = 31, −16, 6, *t* = 3.94, *P* = 0.326, corrected at the non-stationary cluster level). SVCs were used for regions with an *a priori* hypothesis, and significant positive correlations were found in the right middle frontal gyrus (Figs. 1c, 2c, 3c).

**Tables 4** **,** **5** **,** and **6** show all of the significant results.

**Table 4:** Brain regions with significant negative correlations between regional gray matter volume (rGMV) and empathizing {Whole-brain analysis (voxel-level FDR correction, >5 voxels)}.

Area		x	y	z	*t* score	Corrected *P* value (FDR)	Cluster size (mm^3^)
Superior frontal gyrus (B)/Medial frontal Gyrus/middle frontal gyrus(B)/Inferior frontal gyrus (L)/Anterior cingulate gyrus		−21	53	20	4.86	0.011	24611
–Anterior cingulate gyrus							54
–Medial frontal gyrus							4613
–Inferior frontal gyrus	L						45
–Middle frontal gyrus	L						5324
–Superior frontal gyrus	L						7426
–Middle frontal gyrus	R						571
–Superior frontal gyrus	R						2362
Precuneus		3	−57	61	4.13	0.014	551
Orbital frontal gyrus	R	34	28	−26	3.81	0.023	342
Superior parietal lobule	R	10	−76	67	3.58	0.033	106
Precuneus		12	−46	76	3.51	0.037	28
Superior parietal lobule	R	38	−72	59	3.50	0.038	49
Superior medial frontal gyrus		4	32	40	3.48	0.038	19
Anterior cingulate gyrus		−7	35	16	3.44	0.041	45

Empathizing was negatively correlated with rGMV of various areas in the prefrontal cortex, anterior cingulate gyrus, right superior parietal lobule, and precuneus.

Abbreviations: B, bilateral; EQ, empathizing quotient; FDR, false discovery rate; L, left; R, right; SQ, systemizing quotient; SVC, small volume correction.

**Table 5:** Brain regions with significant positive correlations between regional gray matter volume (rGMV) and systemizing.

Area		x	y	z	*t* score	Corrected *P* value (FDR)
ROI analysis (SVC)						
Middle frontal gyrus	R	48	48	30	3.46	0.029

Systemizing was positively correlated with rGMV of the right middle frontal gyrus.

Abbreviations: FDR, false discovery rate; R, right; ROI, region of interest; SVC, small volume correction.

**Table 6:** Brain regions with significant negative correlations between regional gray matter volume (rGMV) and systemizing {Whole-brain analysis (non-stationary cluster size)}.

Area		x	y	z	*t* score	Corrected *P* value
Insula/putamen	L	−31	−3	15	3.71	0.026

Systemizing was negatively correlated with rGMV of an anatomical cluster including the left insula and left putamen.

Abbreviations: L, left.

### Correlation between rGMV and D Scores Across Sexes

Multiple regression analysis, including age, sex, general intelligence, and total brain volume, showed that the D score was significantly and positively correlated with rGMV in a large anatomical cluster that included regions of the bilateral mPFCs, bilateral superior frontal gyrus, middle frontal gyrus, left IFG and regions of the precuneus, middle cingulate gyrus, anterior cingulate gyrus, right superior parietal lobule, and right extranuclear area close to the caudate, thalamus, and right temporal pole (Figs. 1a, 2a, 3a). **Table 7** shows all of these results. No significant negative correlation was observed between rGMV and the D score in the whole-brain analysis.

**Table 7:** Brain regions with significant positive correlations between regional gray matter volume (rGMV) and the D score { Whole-brain analysis (voxel-level FDR correction, >5 voxels)}.

Area		x	y	z	*t* score	Corrected*P* value	Cluster size(mm^3^)	Regions with a negativecorrelation with EQ	Regions with a positivecorrelation with SQ
Superior frontal gyrus (B)/Medial frontal Gyrus/middle frontal gyrus (B)/Inferiorfrontal gyrus (L)/Anterior cingulate gyrus		−12	61	6	5.02	0.009	38316		
–Anterior cingulate gyrus							952	99.5	96.3
–Medial frontal gyrus							6696	100	0
–Inferior frontal gyrus	L						144	99.3	0
–Middle frontal gyrus	L						6304	100	0
–Superior frontal gyrus	L						9520	100	0
–Middle frontal gyrus	R						3648	99.8	76.0
–Superior frontal gyrus	R						6688	100	0
Precuneus		3	−56	63	3.97	0.015	3383	100%	76.1%
Middle cingulate gyrus	R	15	−2	36	3.66	0.024	133	99.2%	99.2%
Anterior cingulate gyrus		−3	34	19	3.62	0.026	863	99.9%	0%
Superior parietal lobule	R	38	−75	58	3.58	0.027	247	99.6%	0%
Inferior frontal gyrus	L	−62	24	6	3.32	0.039	6	100%	83.3%
Superior medial frontal gyrus		6	35	41	3.31	0.039	32	100%	0%
Inferior frontal gyrus	L	−57	22	29	3.21	0.045	7	100%	0%
Extra-nuclear		12	0	4	3.19	0.046	12	0%	100%
Temporal pole	R	37	8	−28	3.17	0.048	6	83.3%	83.3%

The D score was positively correlated with rGMV of various areas in the prefrontal cortex, anterior cingulate gyrus, right superior parietal lobule, precuneus, right extra-nuclear area, and right temporal pole.

Abbreviations: B, bilateral; EQ, empathizing quotient; FDR, false discovery rate; L, left; R, right; SQ, systemizing quotient.

SVC detected significant negative correlations in the right STS in areas with a strong *a priori* hypothesis that lacked significance in the whole-brain analysis (**Table 8**). A trend-level negative correlation was also found between rGMV and the D score in an anatomical cluster that included the left posterior insula and left putamen (*x, y, z* = −28, −11, 5, *t* = 3.85, *P* = 0.061, corrected at the non-stationary cluster level), and significant correlations were observed between rGMV and the SQ score, as described below.

**Table 8:** Brain regions with significant negative correlations between regional gray matter volume (rGMV) and the D score.

Area		x	y	z	*t* score	Corrected *P* value (FDR)	Cluster size (mm^3^)	Regions with a positive correlation with EQ	Regions with a negative correlation with SQ
ROI analysis (SVC)									
Superior temporal sulcus	R	47	−63	11	3.85	0.012	1303	74.4	96.5

The D score was negatively correlated with rGMV of the right superior temporal sulcus.

Abbreviations: EQ, empathizing quotient; FDR, false discovery rate; R, right; ROI, region of interest; SQ: systemizing quotient; SVC: small volume correction.

Because the D score is the difference between S and E, rGMV correlates of the D score are likely to be also correlated with one or both SQ and EQ scores. To reveal how the relationship between rGMV and the EQ and/or SQ score contributed to the significant relationship between the D score and rGMV, we performed the following procedures: (1) We created mask images for each significant cluster of the relationship between the D score and rGMV (described in **Table 8** **)**. (2) We applied these mask images to the whole-brain multiple regression analysis of EQ/SQ (the same multiple regression analysis model described above). (3) We investigated whether EQ and/or SQ scores showed significant relationships with rGMV using SVC and FDR in the areas of the mask images where significant clusters showing a correlation between rGMV and the D score were found. The largest of these clusters was a cluster in the anterior part of the brain (the uppermost cluster in **Table** **7**) that did not appear to be a homogeneous area. Therefore, we subdivided this cluster into seven major areas (areas that belonged to the right/left middle/superior frontal gyrus, anterior cingulate gyrus, medial frontal gyrus, and left IFG) before the analyses. To determine the anatomical areas used in these analyses, we used the WFU PickAtlas Tool (http://www.fmri.wfubmc.edu/cms/software#PickAtlas) [65], [66] with the Talairach Daemon [67] option. This method was also used to divide the large cluster in the analyses described in subsection 3.4.

The significant positive correlation between rGMV in the right extra-nuclear area close to the caudate and the D score was formed by the correlation between rGMV in this area and the SQ score. A significant negative correlation between rGMV in the right STS and the D score and significant positive correlations between rGMV in the anterior cingulate gyrus and right middle frontal gyrus in the largest anterior cluster, in the precuneus and middle cingulate gyrus, in one of the two clusters in the left IFG, and in the right temporal pole and the D score were formed by both of positive correlations between rGMV and the SQ score and negative correlations between rGMV and the EQ score The significant correlations between rGMV and the D score in all of the other areas were formed by negative correlations between rGMV and the EQ score.

### Interaction Effects between Sex and EQ/SQ/D Score on rGMV in both Sexes

ANCOVAs using data from both sexes detected no interaction effects between (a) the EQ score and sex on rGMV; (b) between the SQ score and sex on rGMV; and (c) between the D score and sex on rGMV.

## Discussion

This study investigated rGMV correlates of empathizing and systemizing and their sex-related differences in a large sample of typically developing young adults. We aimed to clarify the following unresolved issues: (a) integration of previously reported inconsistent findings of rGMV correlates of empathy/empathizing using a large sample; (b) identification of localized anatomical correlates of systemizing; and (c) identification of sex-related differences in rGMV correlates of empathizing and systemizing. We also investigated these issues for D scores.

### Summary

In agreement with our hypothesis, rGMVs in regions in the DMN were correlated with empathizing, while those in EAS regions were correlated with systemizing. Further, consistent with the previously suggested link between the mirror neuron system (MNS) and empathy, rGMVs in part of MNS were correlated with empathizing. In agreement with a previous study, systemizing was negatively correlated with rGMV in a region around the left posterior insula and left putamen and positively correlated with rGMV in the middle cingulate gyrus. The D score was correlated with rGMV in most of these areas. In addition to our other significant results, these findings reconciled with the findings of previous analyses of rGMV correlates of empathizing/empathy in typically developing young adults and localized rGMV correlates of empathizing, systemizing, and the D score. The significant results that agreed with our hypothesis showed that empathizing was generally negatively correlated with rGMV in these DMN areas, whereas systemizing was positively correlated with rGMV in these EAS areas. Several regions exhibiting a significant correlation between rGMV and the D score, a positive correlation between rGMV and systemizing and a negative correlation between rGMV and empathizing were observed simultaneously (in the same regions), suggesting an overlap between these two despite the low correlation between empathizing and systemizing. This overlap suggests the involvement of developmental or cognitive mechanisms, as discussed later, and this might be in line with the suggestion that empathizing and systemizing compete neurally in the brain [11]. Finally, rGMV correlates of empathizing included areas close to the midline and also extended well into the lateral parts of the brain, thus covering DLPFCs. We therefore integrated most previously reported inconsistent findings of the rGMV correlates of empathy/empathizing. We identified several localized anatomical correlates of empathizing, systemizing, and the D score in EAS, DMN, and MNS areas. We did not find sex-related differences in rGMV correlates of empathizing, systemizing, or the D score.

### Comparison of Our Results Regarding Empathizing with Our Hypothesis and with Previous Study Results and Functions of Identified Areas

Areas showing significant correlations between (a) rGMV and empathizing or (b) significant correlations between rGMV and the D score (formed by a correlation between rGMV and empathizing) were largely consistent with previous reports and our hypothesis and the previously suggestion that that MNS plays a key role in empathy. These areas involve parts of DMN (mPFC, orbital frontal gyrus, precuneus, right STS, and right temporal pole), MNS (left IFG and right posterior parietal lobule), and the anterior cingulate gyrus. The mPFC, orbital frontal gyrus, precuneus, STS, and right temporal pole are robust DMN regions [21], [68] and are key nodes in areas related to social cognition [69]. The mPFC and contingent regions are involved in assessing the psychological attributes of a person, regardless of whether this is self [22], [70] or non-self [22]. Several studies have shown that the precuneus is involved in perspective taking [71], which plays a key role in empathy [3]. STS plays a key role in the perception of social signals [72], and the regional GM structure of the right STS is positively correlated with the skill of processing social cues [73] and with interpersonal emotional intelligence [74]. The main function of the right temporal pole is to link high-level sensory representations with emotional responses and social memory [75]. The right temporal pole is involved in empathy- and TOM-related tasks [76] and in other tasks that require the consideration of another individual’s thoughts and emotions, possibly via the utilization of this basic function [75]. These regions may facilitate empathizing through these functions. An area in mPFC was identified as an anatomical correlate of empathizing by Cheng et al. [39], and lesion studies have consistently detected an association between this area and empathy, as described earlier. An area in the left frontal gyrus and precuneus was identified as an rGMV correlate of a subfactor in another questionnaire related to empathy [77]. In the present study, rGMV in the precuneus was negatively correlated with an empathic concern subfactor, i.e., the tendency to experience feelings of sympathy and compassion for unfortunate individuals. The rGMV and WM structure of the right STS are negatively correlated with ASQ, which evaluates social and communication skills, and may well be related to empathizing and other symptoms of ASCs [44], [64]. The right temporal pole has not been correlated with empathy scales in normal samples, but atrophy of this region has been associated with a lack of empathy in clinical patients [25]. The left IFG and right superior parietal lobule are parts of MNS [78]. The more anterior regions of the posterior parietal lobule are often considered as MNS, but the superior parietal lobule area is also included in MNS and responds to the action of a movement [78]. The involvement of these areas in empathizing is consistent with the widely held view that MNS facilitates understanding of the intentions of others and plays important roles in empathy and empathic dysfunction in ASCs [79], [80]. The correlation between rGMV in the left IFG/right posterior parietal cortex and empathizing agrees with a previously reported correlation between rGMV in IFG and empathic concern [77]; a correlation between rGMV in IFG and empathizing in children [41]; a correlation between rGMV in the right parietal lobule, including the superior posterior lobule, and empathy scales [39]; and clinical studies that have linked empathic dysfunction with lesions in IFG [24]. Previous studies of rGMV and empathy, such as the study of Banissy et al. (2012), detected an association between high rGMV and dACC. dACC is believed to play a central role in empathy because it is consistently active when perceiving the pain of others and is related to the unpleasantness of pain [81], [82]. The brain structure in this area may relate to empathizing via this function, although there are other possibilities. This area has a wide range of functions, although its underlying function is considered to be negative surprise [83]. Higher rGMV in this area is associated with several conditions related to negative emotions such as anxiety and fear in normal samples [84]–[86]. Low rGMV in this area was also associated with a higher quality of life (QOL) in our previous study [61]. Furthermore, it is well known that positive emotions are strongly associated with empathy [87]. This association was confirmed in our study where there was a robust correlation between QOL [88] and empathizing (r = 0.299, *P* = 5.51*10^−12^, simple regression analysis; both datasets were obtained from 509 subjects). Low rGMV in this area may result in fewer negative emotions, which may lead to greater empathizing. However, we could not find rGMV correlates of empathizing in the insula despite three previous GM studies [41]–[43] and our own previous WM study having identified anatomical correlates in this area [89]. For a related discussion on possible reasons for these discrepancies, see the two paragraphs below.

### Comparison of Our Results Regarding Systemizing with Our Hypothesis and Previous Study Results and Functions of Identified Areas

Areas with significant correlations between (a) rGMV and systemizing and (b) rGMV and the D score (formed by the correlation between rGMV and systemizing) observed in the present study that were consistent with our hypothesis and with those of other studies included areas in EAS, such as the right middle frontal gyrus, dorsal part of the anterior cingulate gyrus, and middle cingulate gyrus, and an anatomical cluster that included part of the basal ganglia (putamen) and the posterior insula. rGMV in an anatomical cluster that included the dorsal part of the anterior to the middle cingulate cortex was positively correlated with systemizing and the D score, while rGMV in a cluster around the architecture of the basal ganglia was negatively correlated with systemizing and the D score in a previous study of empathizing and systemizing [40]. We here utilized different statistical methods and successfully identified more localized rGMV correlates of systemizing/D score. Unlike a previous study [40], a dissociation was observed in the correlation with systemizing/D score between the putamen (negative correlation) and a region around the caudate (positive correlation). This could have been due to a difference such as the use of a more conservative voxel-level threshold. The right middle frontal gyrus and dACC are robust parts of EAS [21]. This part of the middle frontal gyrus is DLPFC, which is involved in the manipulation or mental operation of objects retained in the mind of an individual [90]. dACC is involved in the prediction, monitoring, and detection of errors, possibly via the aforementioned main function of this region, i.e., negative surprise [83]. The middle cingulate gyrus has an important role in tracking higher-order probabilistic statistics related to the environment [91]. The caudate is not part of the resting-state functional connectivity network involved with DLFPC [21], although this area is usually coactive with DLPFC during a wide range of tasks [92]. The area is considered to be deeply involved in learning, memory, and feedback processing [93]. These functions may allow these regions to support the analysis of a system in terms of the rules that govern the system in order to predict its behavior. Conversely, the posterior insula and putamen are involved with distressing emotions such as hatred [94], romantic love [95], disgust [96], and anger [97], [98], which drive an individual to think and behave according to these emotions [94], [99]. The negative correlation between rGMV and systemizing in this area may reflect less distressing emotions in subjects with higher systemizing, which may make it easier to observe objects without these distressing emotions and to analyze the system logically rather than emotionally. In agreement with this notion, systemizing has been reported to be consistently negatively correlated with neuroticism [100], which is the tendency to experience distressing feelings [101].

### Discrepancies between the Present Results and Our Hypothesis/Expectations and Previous Study Results

Some discrepancies were observed between the present results and our hypothesis/expectations and previous study results. These are classified as follows: (a) differences in the positivity or negativity of correlations between studies; (b) differences in the positivity or negativity of correlations between regions; (c) differences in the positivity or negativity of correlations between the different types of cognitive functions; (d) overlap between rGMV correlates of systemizing and empathizing.

Differences in the positivity or negativity of correlations between different studies may have arisen because lower rGMV reflects advanced cortical development and better functioning in some regions in typically developing young adults, but neuronal degeneration or advanced aging in older adults and clinical samples, immature development in small children, and worse functioning in these populations. Significant negative correlations between empathizing and rGMV were found in the mPFC, precuneus, right temporal pole, left IFG, and right superior parietal lobule. As discussed earlier, we interpreted lower rGMV as indicative of higher functioning in these regions. The negative correlations between rGMV and empathy measures in the left IFG and precuneus and their interpretations are consistent with those observed in a previous study of young adults [77]. Indeed, the association between better social or self-related cognitive functioning and lower rGMV in regions around areas close to mPFC has been well demonstrated in young adults [74], [102]. In other cases, cortical thinning was associated with greater or increased cognitive functioning [103]. In clinical and older samples, however, positive correlations were found between empathy measures and rGMV in the mPFC, IFG, posterior parietal lobule, and right temporal pole [25]. As discussed previously, the cortices exhibit developmental thinning after adolescence [104]. Thinned cortices are thought to represent advanced development in young adult samples with better functioning [74], [77], [102] but may represent the loss of synapses and neurons and degeneration in older or clinical samples [105], [106]. In small children who have not reached the age of cortical thinning, lower rGMV may reflect delayed development. These differences in physiological mechanisms underlying individual differences in GMV in different populations may explain the aforementioned differences in the positivity or negativity of correlations between different studies. However, as discussed previously [60], [74], regions around STS and the posterior insula do not show developmental cortical thinning [104], and in these regions, the link between greater cortical thinning and advanced development was not supported. Thus, the present results together with those of previous studies of young adults and children support the interpretations for these areas [41], [44], [60], [73]. The lack of the rGMV correlates of empathizing in the anterior insula in the present study differs from previous studies [41]–[43], [89] that have reported such an association and may also be related to this point since these areas show a peak rGMV at around the age of the present study sample [107]. It is also possible that the difference may be explained by statistical fluctuations or the fact that these areas show rGMV reduction not only during development but also in a wide range of psychiatric diseases [108].

As for the difference in the positivity or negativity of the correlation according to the cognitive function type, contrary to the notion above, positive correlations were observed between rGMV and systemizing in areas that exhibited developmental cortical thinning after adolescence, such as the middle frontal gyrus [104]. In cognitively intact younger adults, thicker cortices are associated with facilitated cognitive functioning linked to externally directed attention-demanding tasks. The opposite may be true for social and emotional cognitive functions. Contrary to this hypothesis, however, a negative correlation was observed between cognitive functions and cortical thickness, and studies of intelligence often show that thicker cortices are associated with higher intelligence [109]. On the basis of these studies, greater GMV and thicker cortices are hypothesized to link to more efficient cognitive functioning [110]. In agreement with this hypothesis, our previous study of young adults showed that higher creativity, greater ability to resolve cognitive interferences, and a greater working memory span, which are considered to be externally directed attention-demanding tasks, were associated with more rGM in the lateral PFC [55], [111] (the GM link to the working memory span is from unpublished data). Based on these findings, we suggest that in cognitively intact younger adults (a) cognitive functions such as social and emotional abilities and empathizing are preferentially associated with lower rGMV in relevant brain regions and are possibly increased by synaptic pruning; (b) cognitive functions such as those measured by externally directed attention-demanding tasks (i.e., tasks measured by psychometric intelligence tests) and systemizing are associated with higher rGMV. Alternatively, cognitive functions with more complex and refined information processing may be facilitated by synaptic pruning and thinned cortices [112], while cognitive functions that require a larger capacity, strength, or faster speed may be facilitated by more neurons or synapses and thicker cortices. However, this is simply pure speculation based on imaging study results and more data are required to test this hypothesis. The causes of the positive correlations between rGMV and systemizing (and other related cognitive functions) and negative correlations between rGMV and empathizing (and social and emotional cognitive functions) may be related to autistic traits and a lack of synaptic pruning. Interestingly, autistic subjects, who have a low ability for empathizing and other related cognitive functions but a high ability for systemizing and its related cognitive functions, also have cortices with higher rGMVs in some of the lateral and medial prefrontal and parietal areas [113]. Cortices with higher rGMVs detected in autistic subjects are believed to be related to a lack of synaptic pruning in the brain [114]. Individual differences in empathizing and systemizing and in traits associated with the autistic spectrum are observed even among normal samples [115]. These individual trait differences may be partly due to a lack of synaptic pruning in the brain. This hypothesis may be consistent with the hypothesis that autism and Asperger’s syndrome lie on a continuum where Asperger’s syndrome is a “bridge” between autism and normality [116]. Interestingly, the aforementioned cognitive functions positively correlated with rGMV in young adults (i.e., working memory span, resolution of cognitive interference, and creativity measured by divergent thinking) were not necessarily compromised in higher functioning autistic subjects [117]–[119], although this point may be disputed, while the self-, social-, and emotion-related cognitive functions negatively correlated with rGMV [74] were robustly impaired in individuals with ASCs [120]. However, whether the same neurophysiological mechanisms underlie individual differences in normal and clinical samples is not guaranteed and may be case-specific. Furthermore, some meta-analyses have shown decreased rGMV in some areas in ASCs (especially in the medial temporal structures, which were not included in the present study) and increases in rGMV [113], [121]–[123] and interaction effects between age and ASCs on brain structures [121], [123], [124]. There are discrepancies and congruencies among these meta-analytic studies, and the abovementioned discussions have to be viewed cautiously.

Finally, we suggest a few possible explanations for the overlap of negative/positive rGMV correlates of empathizing and positive/negative rGMV correlates of systemizing. Significant correlations of empathizing and systemizing in areas with an *a priori* hypothesis or areas that had been previously identified as significant were described above. However, there were also overlaps of negative/positive rGMV correlates of empathizing and positive/negative rGMV correlates of systemizing in many areas, particularly the rGMV correlates of empathizing that extended far into the lateral PFCs. This pattern was very consistent with the results of our previous study of empathizing/systemizing and WM volume [89]. The overlaps described above cannot be explained by a negative correlation between SQ and EQ because no such relationship was observed in this study. This contrasts with the results of a previous study where regions belonging to DMN and those belonging to EAS were clearly segregated by functional activity and functional connectivity analyses [21]. However, associations of the GM structures of DMN (especially mPFC) and those of EAS with psychometric intelligence tests (during which only EAS regions are activated [125]) are actually common in structural studies [110]. One possible explanation for this phenomenon from recent findings is related to the development of the brain structure, namely that rGMV in one area has a very strong correlation with rGMV in the contralateral area and contingent areas regardless of the networks to which these contingent areas belong [126]. Given this strong correlation and rGMV co-development in contingent regions, when rGMV in mPFC is correlated with empathizing, it is not surprising that rGMV in DLPFC has a similar correlation with empathizing. Another possibility suggested is that empathizing and systemizing compete neurally in the brain and that the D score provides information on the trade-off between the two [11]. This type of relationship may result from cognitive mechanisms such as higher systemizing that cannot coexist with higher empathizing in some senses [14] or physiological mechanisms such as advanced synaptic pruning that may underlie higher empathizing and lower systemizing, as suggested above. Other possibilities include higher smoothing values, insufficient normalizing, and fundamental individual differences in the precise anatomical locations of certain functions.

### Clinical Implications

As discussed above, the present findings were, in part, congruent with the previous findings of a meta-analytic study of the brain structures of autistic subjects, although there were some apparent discrepancies in the medial temporal structures, as discussed. Thus, the present findings may be, at least in part, in line with the notion that ASCs exist on a continuum with normality [5] and may imply the ASC cognitive patterns that can be explained largely by higher systemizing and lower empathizing [9]. The brain structures of autistic subjects may thus be partly explained by anatomical correlates of systemizing and empathizing. However, as discussed, the remaining incongruencies among the structural studies of empathizing and systemizing in normal subjects, discrepancies among the meta-analytic studies of autistic subjects, may be due to differences in subjects’ characteristics, such as age, which can affect the pattern of anatomical correlates of cognition. Thus, these findings should be considered cautiously until well-controlled direct comparisons of brain structures and of empathizing and systemizing are performed in large samples of normal and autistic subjects.

## Supporting Information

Methods S1 **Supplemental methods.** Supplemental descriptions of details of methods.
(DOCX)

Discussion S1 **Supplemental discussion.** Supplemental discussion regarding limitations of the study.
(DOCX)
