# Supplementary material for: Regional Gray Matter Volume Is Associated with Empathizing and Systemizing in Young Adults
Source: PLoS One. 2014 Jan 7;9(1):e84782. doi: 10.1371/journal.pone.0084782 (PMC3883687; doi:10.1371/journal.pone.0084782)
Supplement: Methods S1 — Supplemental methods. Supplemental descriptions of details of methods. (DOCX) [file pone.0084782.s001.docx]

**Methods S1.** Supplemental methods.

Supplemental descriptions of details of methods.

**Subjects.** Five hundred and sixty-seven healthy, right-handed individuals (329 men and 238 women; mean age, 20.8 years; standard deviation, 1.9) participated in this study as part of an ongoing project investigating associations among brain imaging, cognitive functions, aging, genetics, and daily habits. Data of the study subjects has been used in other studies irrelevant to the theme of this study [[1](#_ENREF_1),[2](#_ENREF_2),[3](#_ENREF_3),[4](#_ENREF_4),[5](#_ENREF_5),[6](#_ENREF_6),[7](#_ENREF_7),[8](#_ENREF_8),[9](#_ENREF_9)]. Some subjects who participated in this study also became the subjects of intervention studies (psychological and imaging data recorded before the interventions were used in this study) [[10](#_ENREF_10),[11](#_ENREF_11),[12](#_ENREF_12)]. The project includes psychological tests and MRI scans irrelevant to this study; thus, these were not described although they were performed together with those described in this study. The psychological measures and MRI scans used varied depending on the timing of the experiment. All subjects were college, university, or postgraduate students or had graduated within the previous year. Most were associated with Tohoku University. They were recruited using advertisements placed on bulletin boards at Tohoku University or via email. The advertisements and emails specified the exclusion criteria including handedness the presence of metal in or around the body, claustrophobia, the use of certain drugs, a history of certain psychiatric or neurological diseases, and previous participation in related experiments. We provided questionnaires to all potential experimental subjects for assessing psychiatric illnesses and ascertaining recent drug use. None had a history of neurological or psychiatric illness. The assessments were performed during and after recruitment and were based on voluntary self-reports. The study was completed at a single site, and all data were collected at the Institute of Development, Aging and Cancer, Tohoku University.

**Reasons for not analyzing multiple factors associated with EQ and SQ.** We did not analyze the multiple factors associated with EQ and SQ [14] because such analyses cannot provide relevant information on the findings of previous studies, which have almost always used total EQ and SQ scores. Furthermore, the structure of factors is usually unstable and dependent on subjects and statistical methods [[13](#_ENREF_13)].

**Rationale for the choice of models for imaging analyses.** One may recommend comparing sex-related differences in rGMV (investigation of areas where males or females show larger rGMV) with the rGMV correlates of the EQ/SQ/D scores. However, we decided to omit this analysis because the manuscript was already too long and it would take up too much space to describe this analysis accurately. It is also irrelevant to our purpose, and such descriptions would have severely disrupted the integrity of the study under our inadequate descriptions. Our previous study in children completely denied any such associations, and our results point to the same conclusion [[14](#_ENREF_14)].

One may also suggest dividing rGMV by the total brain volume instead of including this volume as a covariate in the statistical model because this may avoid the problem of multicolinearity associated with the high correlation between sex and the total brain volume. However, although we appreciate this method as an attempt to correct for the effects of the total brain volume, we believe it cannot prevent the problems caused by multicolinearity. One such problem is the change in the sign (+ or −) of the regression coefficient; we can easily see that these changes in signs occur regardless of whether the total brain volume is the covariate or the dependent variable is rGMV/total brain volume. In our opinion and in this case, to the best of our knowledge, these changes were not problematic for what we were examining in these analyses. Thus, we followed the widely used method of correcting for global effects (including the total brain volume as a covariate).

**Details of the cluster size tests used and the rationale for the choice of the present thresholding methods for imaging analyses.** The statistical significance level was set at *P <* 0.05, corrected at the non-isotropic adjusted cluster level [[15](#_ENREF_15)] with an underlying voxel level of *P <* 0.0025. In this non-isotropic cluster size test of random field theory, a relatively high cluster-determining threshold combined with high smoothing values of more than six voxels leads to appropriate conservativeness with real data. With high smoothing values, an uncorrected threshold of *P* < 0.01 seems to lead to anticonservativeness, whereas that of *P* < 0.001 seems to lead to slight conservativeness [[16](#_ENREF_16)]. We used a cluster size test because of its sensitivity [[17](#_ENREF_17)]. Small structures such as the amygdala may be excluded if cluster size tests are used, but our hypothesis did not include such structures. Furthermore, additional analyses did not identify any significant results in small structures such as the amygdala when a voxel-level family-wise error correction was used. Despite the strength of this correction, previous studies have suggested that it tends to eliminate false and true positives if it is applied to entire neuroimaging datasets [[18](#_ENREF_18),[19](#_ENREF_19)]. To the best of our knowledge, however, a pure voxel-level family-wise error correction (without a small volume correction) has rarely been used in VBM studies to investigate correlations between traits or personalities and GM structures in normal subjects [e.g., [20](#_ENREF_20),[21](#_ENREF_21),[22](#_ENREF_22),[23](#_ENREF_23),[24](#_ENREF_24),[25](#_ENREF_25),[26](#_ENREF_26),[27](#_ENREF_27)]. We believe that when there are no special circumstances cluster tests should be chosen because they have superior sensitivity compared with only the height threshold is used to determine the significance [[17](#_ENREF_17)]. Therefore, we chose the cluster size test in most contrasts.

However, this test also has a weakness. It does not indicate exactly where the effect of interest is located within a cluster. Thus, it is not suitable when a cluster is very large. Two contrasts in this study, namely the analyses testing the positive correlation between rGMV and the D score and the negative correlation between rGMV and the EQ score, held true, and significant large clusters were identified. In these contrasts, the same results were observed when a more conservative cluster-determining threshold, such as *P* < 0.001, uncorrected, was used. In these contrasts, a multiple comparison correction was performed using the FDR approach [[19](#_ENREF_19)] as the second-best option. Only clusters that contained more than five voxels below the threshold of *P* < 0.05, corrected for FDR, were reported.

**Supplemental Discussion**

**Limitations**

This study has several limitations. A lack of statistical power might have prevented the detection of significant results in other regions. Our sample size was very large for this type of study. However, some results were only marginally significant when sensitive statistical methods were used. This may have been because we could not cover all of the previous findings related to systemizing/empathizing (empathy), such as the rGMV correlates of systemizing in the left posterior cortex of children [[14](#_ENREF_14)], which were consistent with our hypothesis, and possibly because of the false negatives caused by the lack of statistical power. However, a number of methodological differences (especially subject characteristics) may also have contributed. Another fundamental limitation of these types of cross-sectional whole-brain analyses is that they cannot demonstrate causal relationships. Certain types of training can alter brain structures [[28](#_ENREF_28),[29](#_ENREF_29)]. Thus, a number of possible mechanisms might form associations between empathizing–systemizing and rGMV. For example, if empathizing and systemizing are related to certain behaviors that can alter brain structures, neural mechanisms underlying increased or decreased rGMV might lead to increased empathizing–systemizing. Limited sampling of the full range of intellectual abilities is a common hazard when sampling from college cohorts. Whether our findings would also hold across the full range of population samples and normal distribution must be determined with larger and more representative samples. However, university students (the samples in this study) and the general population have been shown to have equivalent EQ and SQ scores [[30](#_ENREF_30)]. Thus, any deviations in the EQ and SQ scores from the scores for the general population should not be a concern. Finally, we stipulated a specific age range. Subjects of different ages should have different function–structure relationships. Thus, while this may be one of the study limitations, it is also a strength because it reveals the anatomical correlates of cognitive functions. However, how the relationships between empathizing/systemizing/D score differ among subjects of different ages, such as the elderly, remains to be investigated. Finally, since we did not perform official, prolonged diagnostic procedures for all the existing diseases to exclude subjects with existing diseases, it was possible to include some subjects who had never been diagnosed with certain diseases but could be diagnosed with certain diseases in the recent medicine if they went to the hospital. Even if the subjects were not 100% fit, the diseases that they might have suffered from were not sufficiently alarming to require a hospital visit. Furthermore, the most prevalent of the psychiatric diseases seem to be mood disorders, anxiety disorders, social phobia, and PTSD. Patients with these psychiatric diseases are not likely to participate in complex procedures such as MRI experiments. This type of bias may be a limitation of almost all imaging studies of this type that have assessed non-clinical samples.

**References**

1. Takeuchi H, Taki Y, Hashizume H, Sassa Y, Nagase T, et al. (2011) Failing to deactivate: the association between brain activity during a working memory task and creativity. Neuroimage 55: 681-687.

2. Takeuchi H, Taki Y, Sassa Y, Hashizume H, Sekiguchi A, et al. (2010) Regional gray matter density associated with emotional intelligence: evidence from voxel-based morphometry. Human Brain Mapping: Epub ahead of print.

3. Takeuchi H, Taki Y, Sassa Y, Hashizume H, Sekiguchi A, et al. (2010) White matter structures associated with creativity: Evidence from diffusion tensor imaging. Neuroimage 51: 11-18.

4. Takeuchi H, Taki Y, Sassa Y, Hashizume H, Sekiguchi A, et al. (2010) Regional gray matter volume of dopaminergic system associate with creativity: Evidence from voxel-based morphometry Neuroimage 51: 578-585.

5. Takeuchi H, Taki Y, Hashizume H, Sassa Y, Nagase T, et al. (2011) Cerebral blood flow during rest associates with general intelligence and creativity. PLoS ONE 6: e25532.

6. Takeuchi H, Taki Y, Sassa Y, Hashizume H, Sekiguchi A, et al. (2011) Verbal working memory performance correlates with regional white matter structures in the fronto-parietal regions. Neuropsychologia 49: 3466-3473

7. Takeuchi H, Taki Y, Sassa Y, Hashizume H, Sekiguchi A, et al. (2011) Regional gray matter density associated with emotional intelligence: Evidence from voxel-based morphometry. Human Brain Mapping 32: 1497-1510.

8. Takeuchi H, Taki Y, Sassa Y, Hashizume H, Sekiguchi A, et al. (2012) Regional gray and white matter volume associated with Stroop interference: Evidence from voxel-based morphometry. Neuroimage 59: 2899-2907.

9. Takeuchi H, Taki Y, Hashizume H, Sassa Y, Nagase T, et al. (2012) The Association between Resting Functional Connectivity and Creativity. Cerebral Cortex Epub ahead of print.

10. Takeuchi H, Taki Y, Sassa Y, Hashizume H, Sekiguchi A, et al. (2011) Working memory training using mental calculation impacts regional gray matter of the frontal and parietal regions. PLoS ONE 6: e23175.

11. Takeuchi H, Taki Y, Hashizume H, Sassa Y, Nagase T, et al. (2011) Effects of training of processing speed on neural systems. Journal of Neuroscience 31: 12139-12148.

12. Takeuchi H, Taki Y, Nouchi R, Hashizume H, Sekiguchi A, et al. (in press) Effects of working memory-training on functional connectivity and cerebral blood flow during rest. Cortex.

13. Takeuchi H, Taki Y, Sassa Y, Hashizume H, Sekiguchi A, et al. (2013) Brain structures associated with executive functions during everyday events in a non-clinical sample. Brain Structure and Function 218: 1017-1032.

14. Sassa Y, Taki Y, Takeuchi H, Hashizume H, Asano M, et al. (2012) The correlation between brain gray matter volume and empathizing and systemizing quotients in healthy children. Neuroimage 60: 2035-2041.

15. Hayasaka S, Phan KL, Liberzon I, Worsley KJ, Nichols TE (2004) Nonstationary cluster-size inference with random field and permutation methods. Neuroimage 22: 676-687.

16. Silver M, Montana G, Nichols TE (2010) False positives in neuroimaging genetics using voxel-based morphometry data. Neuroimage 54: 992-1000.

17. Friston KJ, Holmes A, Poline JB, Price CJ, Frith CD (1996) Detecting activations in PET and fMRI: levels of inference and power. NeuroImage 4: 223-235.

18. Yamasue H, Abe O, Suga M, Yamada H, Inoue H, et al. (2008) Gender-common and-specific neuroanatomical basis of human anxiety-related personality traits. Cerebral Cortex 18: 46-52.

19. Genovese CR, Lazar NA, Nichols T (2002) Thresholding of statistical maps in functional neuroimaging using the false discovery rate. Neuroimage 15: 870-878.

20. Wright CI, Feczko E, Dickerson B, Williams D (2007) Neuroanatomical correlates of personality in the elderly. Neuroimage 35: 263-272.

21. Yamasue H, Abe O, Suga M, Yamada H, Rogers MA, et al. (2008) Sex-linked neuroanatomical basis of human altruistic cooperativeness. Cerebral Cortex 18: 2331-2340.

22. Kaasinen V, Maguire RP, Kurki T, Br ck A, Rinne JO (2005) Mapping brain structure and personality in late adulthood. Neuroimage 24: 315-322.

23. Matsui M, Yoneyama E, Sumiyoshi T, Noguchi K, Nohara S, et al. (2002) Lack of self-control as assessed by a personality inventory is related to reduced volume of supplementary motor area. Psychiatry Research: Neuroimaging 116: 53-61.

24. Blankstein U, Chen JYW, Mincic AM, McGrath PA, Davis KD (2009) The complex minds of teenagers: Neuroanatomy of personality differs between sexes. Neuropsychologia 47: 599-603.

25. Spampinato MV, Wood JN, De Simone V, Grafman J (2009) Neural Correlates of Anxiety in Healthy Volunteers: A Voxel-Based Morphometry Study. Journal of Neuropsychiatry and Clinical Neurosciences 21: 199.

26. Cohen MX, Schoene-Bake JC, Elger CE, Weber B (2008) Connectivity-based segregation of the human striatum predicts personality characteristics. Nature Neuroscience 12: 32-34.

27. Iidaka T, Matsumoto A, Ozaki N, Suzuki T, Iwata N, et al. (2006) Volume of left amygdala subregion predicted temperamental trait of harm avoidance in female young subjects. A voxel-based morphometry study. Brain Research 1125: 85-93.

28. Draganski B, Gaser C, Busch V, Schuierer G, Bogdahn U, et al. (2004) Neuroplasticity: Changes in grey matter induced by training. Nature 427: 311-312.

29. Takeuchi H, Sekiguchi A, Taki Y, Yokoyama S, Yomogida Y, et al. (2010) Training of Working Memory Impacts Structural Connectivity. Journal of Neuroscience 30: 3297-3303.

30. Wakabayashi A, Baron-Cohen S, Uchiyama T, Yoshida Y, Kuroda M, et al. (2007) Empathizing and systemizing in adults with and without autism spectrum conditions: cross-cultural stability. Journal of Autism and Developmental Disorders 37: 1823-1832.
