# Supplementary material for: Regional Gray Matter Volume Is Associated with Empathizing and Systemizing in Young Adults
Source: PLoS One. 2014 Jan 7;9(1):e84782. doi: 10.1371/journal.pone.0084782 (PMC3883687; doi:10.1371/journal.pone.0084782)
Supplement: Discussion S1 — Supplemental discussion. Supplemental discussion regarding limitations of the study. (DOCX) [file pone.0084782.s002.docx]

**Discussion S1.** Supplemental Discussion.

Supplemental discussion regarding limitations of the study**.**

**Limitations**

This study has several limitations. A lack of statistical power might have prevented the detection of significant results in other regions. Our sample size was very large for this type of study. However, some results were only marginally significant when sensitive statistical methods were used. This may have been because we could not cover all of the previous findings related to systemizing/empathizing (empathy), such as the rGMV correlates of systemizing in the left posterior cortex of children [[1](#_ENREF_1)], which were consistent with our hypothesis, and possibly because of the false negatives caused by the lack of statistical power. However, a number of methodological differences (especially subject characteristics) may also have contributed. Another fundamental limitation of these types of cross-sectional whole-brain analyses is that they cannot demonstrate causal relationships. Certain types of training can alter brain structures [[2](#_ENREF_2),[3](#_ENREF_3)]. Thus, a number of possible mechanisms might form associations between empathizing–systemizing and rGMV. For example, if empathizing and systemizing are related to certain behaviors that can alter brain structures, neural mechanisms underlying increased or decreased rGMV might lead to increased empathizing–systemizing. Limited sampling of the full range of intellectual abilities is a common hazard when sampling from college cohorts. Whether our findings would also hold across the full range of population samples and normal distribution must be determined with larger and more representative samples. However, university students (the samples in this study) and the general population have been shown to have equivalent EQ and SQ scores [[4](#_ENREF_4)]. Thus, any deviations in the EQ and SQ scores from the scores for the general population should not be a concern. Finally, we stipulated a specific age range. Subjects of different ages should have different function–structure relationships. Thus, while this may be one of the study limitations, it is also a strength because it reveals the anatomical correlates of cognitive functions. However, how the relationships between empathizing/systemizing/D score differ among subjects of different ages, such as the elderly, remains to be investigated. Finally, since we did not perform official, prolonged diagnostic procedures for all the existing diseases to exclude subjects with existing diseases, it was possible to include some subjects who had never been diagnosed with certain diseases but could be diagnosed with certain diseases in the recent medicine if they went to the hospital. Even if the subjects were not 100% fit, the diseases that they might have suffered from were not sufficiently alarming to require a hospital visit. Furthermore, the most prevalent of the psychiatric diseases seem to be mood disorders, anxiety disorders, social phobia, and PTSD. Patients with these psychiatric diseases are not likely to participate in complex procedures such as MRI experiments. This type of bias may be a limitation of almost all imaging studies of this type that have assessed non-clinical samples.

**References**

1. Sassa Y, Taki Y, Takeuchi H, Hashizume H, Asano M, et al. (2012) The correlation between brain gray matter volume and empathizing and systemizing quotients in healthy children. Neuroimage 60: 2035-2041.

2. Draganski B, Gaser C, Busch V, Schuierer G, Bogdahn U, et al. (2004) Neuroplasticity: Changes in grey matter induced by training. Nature 427: 311-312.

3. Takeuchi H, Sekiguchi A, Taki Y, Yokoyama S, Yomogida Y, et al. (2010) Training of Working Memory Impacts Structural Connectivity. Journal of Neuroscience 30: 3297-3303.

4. Wakabayashi A, Baron-Cohen S, Uchiyama T, Yoshida Y, Kuroda M, et al. (2007) Empathizing and systemizing in adults with and without autism spectrum conditions: cross-cultural stability. Journal of Autism and Developmental Disorders 37: 1823-1832.
